# The effect of body position on intraocular pressure in calves

**DOI:** 10.4102/jsava.v89i0.1638

**Published:** 2018-08-20

**Authors:** Başak Kurt, Halil H. Çağatay, Özgür Aksoy

**Affiliations:** 1 Department of Surgery, Kafkas University, Turkey; 2 Department of Ophthalmology, Karsiyaka Eye Hospital, Turkey

## Abstract

Tonometry is one of the basic diagnostic tests used for the diagnosis of glaucoma and uveitis in veterinary ophthalmology. The Icare^®^ Rebound Tonometer which is a new tonometric device has been shown to be useful in a wide range of species. Eyes (*n* = 48) of 24 Simmental and Montafon calves with a mean age of 7.5 weeks (2–16 weeks), male and female, were subjected to intraocular pressure (IOP) measurement using the Icare^®^ Rebound Tonometer with calves standing and in lateral recumbency. The mean IOP was measured as 9.02 ± 2.38 mmHg in the right eye and 9.08 ± 2.55 mmHg in the left eye. No age-related change was found in intraocular pressure of the calves between 2 and 16 weeks of age. No difference in IOP values was observed between Simmental and Montafon calves. Body position had no effect on IOP in calves. The Icare^®^ Rebound Tonometer was shown to be a suitable diagnostic device for IOP measurement in calves.

## Introduction

Tonometry is one of the basic diagnostic tests used in veterinary ophthalmology. It allows one to determine intraocular pressure (IOP) for the diagnosis of glaucoma and uveitis (Knollinger et al. 2005). Although many tonometers with different measuring principles are used in the veterinary field (Gum et al. 1998; Rusanen et al. 2010), none of these measure IOP directly but predict it by utilising the physical properties of the cornea. Rebound tonometry is quick and easy to apply, provides clear readings and there is no need for topical anaesthesia prior to application (Rusanen et al. 2010; Tofflemire et al. 2015). Intraocular pressure is estimated by contact and rebound of a propelled probe on the surface of the cornea. The final result is obtained by the average of six measurement values obtained from the centre of the cornea (Knollinger et al. 2005). Average values of IOP measurements were obtained in studies conducted on many different animal species (Rusanen et al. 2010). However, information on the use of rebound tonometry in calves and cattle is limited (Knollinger et al. 2005).

Apart from the type of tonometer, there are a number of factors that may cause discrepancies in IOP values. The most influential factors on IOP values are sedation and time when measurements are made. Studies with humans and horses reported that head position also affected IOP values (Galin, McIvor & Magruder 1963; Komaromy et al. 2006). Increased IOP values are obtained with episcleral congestion and an increase in episcleral venous pressure when the head is below the level of the heart (Komaromy et al. 2006).

This study aimed to determine the effect of body position on intraocular pressure in calves.

## Materials and methods

Calves (*n* = 24), 2–16 weeks of age, 12 Simmental and 12 Montafon of both sexes, which presented to the surgical clinic of the Veterinary Faculty of Kafkas University for various complaints, but without eye pathology, were subjected to IOP measurement using the Icare^®^ Rebound Tonometer by the same physician. Animals were not sedated or topically anaesthetised. Measurements were carried out when calves were standing and in lateral recumbency. The animals were manually restrained and the IOP values for both eyes were measured initially when the calves were standing. The animals were then placed in lateral recumbency; after 5 minutes when they calmed down, measurements were repeated. The calves were always physically held in the same position, right eyes were examined first taking care not to constrict the jugular vein or apply pressure on the neck region during restraint. Six measurements were made from the central cornea at each position, and the mean calculated IOP value was recorded. All measurements were taken during spring between 09:00 and 11:00.

The difference between the values in both standing and lying positions for the same eye of animals and the difference between the values for the two eyes of the same animal were statistically determined. The data were analysed with General Linear Model (GLM) procedure. The effects of breed on the investigated parameters such as left eye IOP in standing position, right eye IOP in lying position were determined and the differences in animal ages were adjusted with covariance in the model. Differences in the means of parameters were determined using the Tukey test. All calculations and analyses were executed by Statistical Analyses Software (2009).

## Results

No significant difference was observed between IOP values of the right and left eyes while calves were in standing or lying position. The mean IOP values were determined as 9.45 ± 2.76 mmHg (right) and 9.20 ± 1.86 mmHg (left) in the standing position, and 8.58 ± 1.88 mmHg (right) and 8.95 ± 3.14 mmHg (left) in the lying position. The IOP values recorded in different body positions are presented in Table 1, while the mean IOP values obtained according to body position and breed are shown in Table 2. No age-related differences were recorded in the calves (2–16 weeks of age).

**TABLE 1:** Intraocular pressure values.

No.	Breed	Age (weeks)	Sex	Standing position		Lying position	
				Right	Left	Right	Left
1	Simmental	8	♂	8	9	8	8
2	Simmental	8	♂	8	8	6	7
3	Simmental	8	♂	6	7	7	6
4	Simmental	12	♀	7	9	9	8
5	Simmental	8	♀	9	9	9	10
6	Simmental	2	♂	13	11	11	12
7	Simmental	8	♂	5	7	7	6
8	Montafon	2	♂	12	11	5	5
9	Montafon	8	♂	6	9	9	8
10	Montafon	10	♂	16	10	9	11
11	Montafon	6	♂	14	12	7	10
12	Montafon	8	♀	7	6	12	12
13	Montafon	8	♂	11	8	8	9
14	Montafon	10	♀	9	11	9	11
15	Montafon	6	♂	10	8	6	6
16	Montafon	6	♀	9	9	8	7
17	Montafon	14	♀	10	6	10	18
18	Montafon	16	♀	9	10	9	6
19	Montafon	16	♀	11	9	11	12
20	Simmental	4	♀	6	9	7	7
21	Simmental	6	♂	8	14	12	14
22	Simmental	2	♀	12	9	10	8
23	Simmental	2	♂	9	11	7	9
24	Simmental	3	♂	12	9	10	5

Intraocular pressure was measured in mmHg.

♂, male; ♀, female.

**TABLE 2:** Mean intraocular pressure values (mmHg) by breed and body position.

Breed	Standing		Lateral recumbency	
	Right eye	Left eye	Right eye	Left eye
**Simmental**	8.58 ± 0.74	9.33 ± 0.55	8.58 ± 0.54	8.33 ± 0.75
**Montafon**	10.33 ± 0.80	9.08 ± 0.54	8.58 ± 0.57	9.58 ± 1.04

## Discussion

Sedation and topical anaesthesia as two of the many factors such as measurement time (hour), breed, exercise, postural changes, medicines, blood pressure and seasonal changes (Çetin, Pamuk & Yaprakçı 2014; Gum et al. 1998) were eliminated by using the Icare^®^ Rebound Tonometer. Çetin et al. (2014) conducted a study on young buffalo calves and assessed the results by making IOP measurements at the same time (hours) of day. It was reported that in this way they get safer results. Similarly, in our study, the measurements were made at the same time of day and in the same season, and then reference values were created. The mean IOP value in calves’ eyes was determined as 9.05 ± 2.45 mmHg using measurements with an Icare^®^ Rebound Tonometer in the present study. Tofflemire et al. (2015) measured IOP in calves with an average age of 11 weeks using a Tonovet^®^ and reported the mean value as 15.20 ± 5.20 mmHg. There was no evidence that IOP was regularly increased or decreased in the age range that was assessed in the present study. However, when these two studies are taken into account together, IOP in calves is thought to increase with age. When the effect of breed factor on IOP is considered, the study conducted by Tofflemire et al. (2015) on Holstein breed calves is notable. In the present study, Simmental and Montafon breed calves were examined and no statistically significant difference was found between IOP values in these two breeds.

Although the IOP values obtained from calves’ right and left eyes differed, these values were found to be statistically insignificant. Çetin et al. (2014) also found insignificantly different IOP values for the right and left eyes of young buffalo calves using a Tonopen-XL Applanation Tonometer.

There are many studies on the effect of head position on IOP in humans. This effect is believed to depend on changes in episcleral venous pressure in people when changing from sitting or standing to supine or head down inverted positions (Friberg, Sanborn & Weinreb 1987; Galin et al. 1963). A similar effect was reported to occur in horses when their head positions dropped below heart level during feeding (Komaromy et al. 2006). Another study examining the effect of body position on IOP in dogs determined that pressure in the dorsal position was higher than sternal position (Broadwater et al. 2008). Galin et al. (1963) reported a difference of 2 mmHg – 4 mmHg in humans between sitting or lying positions. In the present study, no statistically significant difference was found in calves.

Although not common in calves and cattle, Townsend et al. (2008) reported congenital secondary glaucoma in dairy cattle. This underscores the importance of routine eye examination and IOP measurement in calves.

## Conclusion

The Icare^®^ Rebound Tonometer has proven to be a suitable diagnostic device for IOP measurement in calves. It is well tolerated by calves and requires no sedation or anaesthesia. The mean IOP value obtained by Icare^®^ Rebound Tonometer in healthy eyes of calves at 2–16 weeks of age in lateral recumbency or standing, measured in spring between 09:00 and 11:00, was determined as 9.05 ± 2.45 mmHg.
